# Hysteroscopy metroplasty for partial septate uterus: Just a matter of infertility?

**DOI:** 10.1002/ijgo.70906

**Published:** 2026-02-24

**Authors:** Alessandro Arena, Agnese Virgilio, Camilla Franceschini, Erika Bettiol, Pierandrea De Benedetti, Roberto Sardina, Giulia Cincotta, Eugenia Mantovani, Renato Seracchioli, Paolo Casadio

**Affiliations:** ^1^ Division of Gynaecology and Human Reproduction Physiopathology IRCCS Azienda Ospedaliero – University of Bologna Bologna Italy; ^2^ Department of Medical and Surgical Sciences (DIMEC) University of Bologna Bologna Italy; ^3^ Unit of Gynecology IRCCS Humanitas Research Hospital, Rozzano Milan Italy; ^4^ Department of Gynecological and Obstetric San Luca Hospital Lucca Italy

**Keywords:** hysteroscopy, metroplasty, Müllerian anomalies, quality of life, septate uterus, symptoms relief

## Abstract

**Objective:**

The primary objective of this study is to assess the impact of hysteroscopic metroplasty on symptom relief, including dysmenorrhea, dyspareunia, and menstrual blood flow in women with partial uterine septa (U2a class). Additionally, the study aims to investigate potential correlations between clinical and ultrasonographic characteristics and changes in these symptoms.

**Methods:**

This was a prospective cohort study conducted at a single center, enrolling women who underwent hysteroscopic metroplasty between March 2022 and September 2023. Participants presented with dysmenorrhea, dyspareunia, and abnormal menstrual blood flow and had expressed a desire for pregnancy. Preoperative symptom severity was measured using a visual analog scale, ranging from 0 to 100. Preoperative three‐dimensional transvaginal ultrasonography was performed to assess septal characteristics. A 12‐month follow‐up was conducted to evaluate changes in symptoms postoperatively. Exclusion criteria included alternative causes of pelvic pain, incomplete septum correction necessitating further surgery, and pregnancy or hormonal therapy within 12 months prior to or following the surgery.

**Results:**

A total of 52 symptomatic patients with U2a uterine septa underwent hysteroscopic metroplasty, and data from 44 patients were analyzed according to the inclusion and exclusion criteria. Postoperative assessments revealed a significant reduction in dysmenorrhea (mean ± standard deviation: 50.2 ± 20.5 vs. 33.1 ± 20.4, *P* < 0.001) and dyspareunia (mean ± standard deviation: 41.4 ± 9.5 vs. 28.6 ± 24.5, *P* < 0.028). However, no significant changes were observed in menstrual blood flow.

**Conclusion:**

Hysteroscopic metroplasty in patients with partial uterine septa results in significant symptom relief, particularly for dysmenorrhea and dyspareunia. These findings underscore the potential benefits of metroplasty beyond reproductive outcomes. Further large‐scale studies are necessary to confirm these results.

## 1 INTRODUCTION

Müllerian anomalies represent a heterogeneous spectrum of congenital malformations of the female reproductive system. The uterine septum is responsible for 35% of all identified uterine malformations, with a prevalence of 2–3% among women of reproductive age. These anomalies result from an incomplete resorption of the uterine cavity due to abnormal regression of the Müllerian ducts during embryonic development.^1^

In 2013, the European Society of Human Reproduction and Embryology (ESHRE) and the European Society for Gynecological Endoscopy (ESGE) defined a septate uterus as having a normal external contour but with an internal indentation at the fundal midline that exceeds 50% of the uterine wall thickness. If the septum reaches the internal uterine os, it is classified as a complete uterine septum (Class U2b); otherwise, it is classified as partial (Class U2a).^2^

While complete uterine septa are routinely treated to improve fertility and pregnancy outcomes, the management of partial septa for similar purposes remains controversial, with no clear consensus on its benefits.^3^, ^4^, ^5^ Further, some patients with a septate uterus report symptom such as dysmenorrhea, chronic pelvic pain, and abnormal uterine bleeding. However, the direct correlation between these symptoms and the anatomical anomaly remains uncertain, and the existing literature on these associations is limited, highlighting the need for further research.^6^

In response to these gaps in knowledge, the objective of this study is to evaluate the impact of hysteroscopic metroplasty on dyspareunia, dysmenorrhea, and menstrual blood flow in symptomatic patients with a partial uterine septum. Additionally, the study will examine potential correlations between ultrasonographic and clinical characteristics of the uterine septum and variations in symptoms following surgery. Finally, the study aims to assess whether hysteroscopic metroplasty results in significant lifestyle changes in women undergoing surgical treatment for partial uterine septa.^7^

## 2 MATERIALS AND METHODS

This was a prospective cohort study conducted at a single tertiary care center. The study was performed in the Department of Gynecology and Physiopathology of Human Reproduction, S. Orsola‐Malpighi Hospital, University of Bologna, Italy, a referral center for minimally invasive gynecologic surgery, following the study protocol (n.552/2024/Oss/AOUBo). The data were reported in accordance with the Strengthening the Reporting of Observational Studies in Epidemiology (STROBE) statement and checklist, and all patients provided written informed consent.^8^

### 2.1 Study design and setting

Participants were enrolled between March 2022 and September 2023. We enrolled patients with a sonographic diagnosis of partial uterine septum (Class U2a, ESHRE/ESGE 2013 classification system, defined as having a normal outline with an internal indentation at the fundal midline exceeding 50% of the uterine wall thickness, without reaching the internal cervical os) who were symptomatic, during most of the menstrual cycle, of dysmenorrhea, dyspareunia, and/or heavy menstrual bleeding. Baseline data were collected using a paper‐based questionnaire at the first visit and using the same questionnaire at the 12‐month +/− 4 weeks follow‐up, during which the patient was contacted via telemedicine. The assessment was conducted blinded to symptoms. All eligible participants were included in one study group and underwent the same intervention. All patients provided informed consent for the use of their anonymized data for research purposes.

### 2.2 Participants

Participants were consecutively recruited from all patients presenting with symptomatic U2a partial septa during the study period. During preoperative assessment, patients completed a Visual Analog Scale (VAS) pain questionnaire, with each symptom rated on a scale of 0 to 100.

Inclusion criteria were symptomatic nulliparous women diagnosed with a partial uterine septum who reported significant discomfort (VAS score > 30/100) related to dysmenorrhea, dyspareunia, and/or heavy menstrual bleeding. For heavy menstrual bleeding, a VAS score > 30/100 was considered clinically significant, in line with previous studies.^9^ Eligible women also had a history of infertility, recurrent pregnancy loss, or a desire for pregnancy.

Symptom severity was recorded prospectively using the same VAS questionnaire at the 12‐month follow‐up. All patients were followed for 12 months. Exclusion criteria included gynecological conditions contributing to pelvic pain, such as adenomyosis, endometriosis, or pelvic inflammatory disease, and conditions associated with pelvic pain like inflammatory bowel disease, interstitial cystitis, and pelvic floor dysfunction. Patients who underwent hormonal therapy within 1 year before or during the follow‐up period, or those who had previous uterine surgery, were also excluded. Additionally, women with ongoing pregnancies during follow‐up were excluded to prevent pregnancy‐related effects on study outcomes. These exclusion criteria were established to ensure the clarity and reliability of the study results.

### 2.3 Preoperative assessment

**FIGURE 1:**
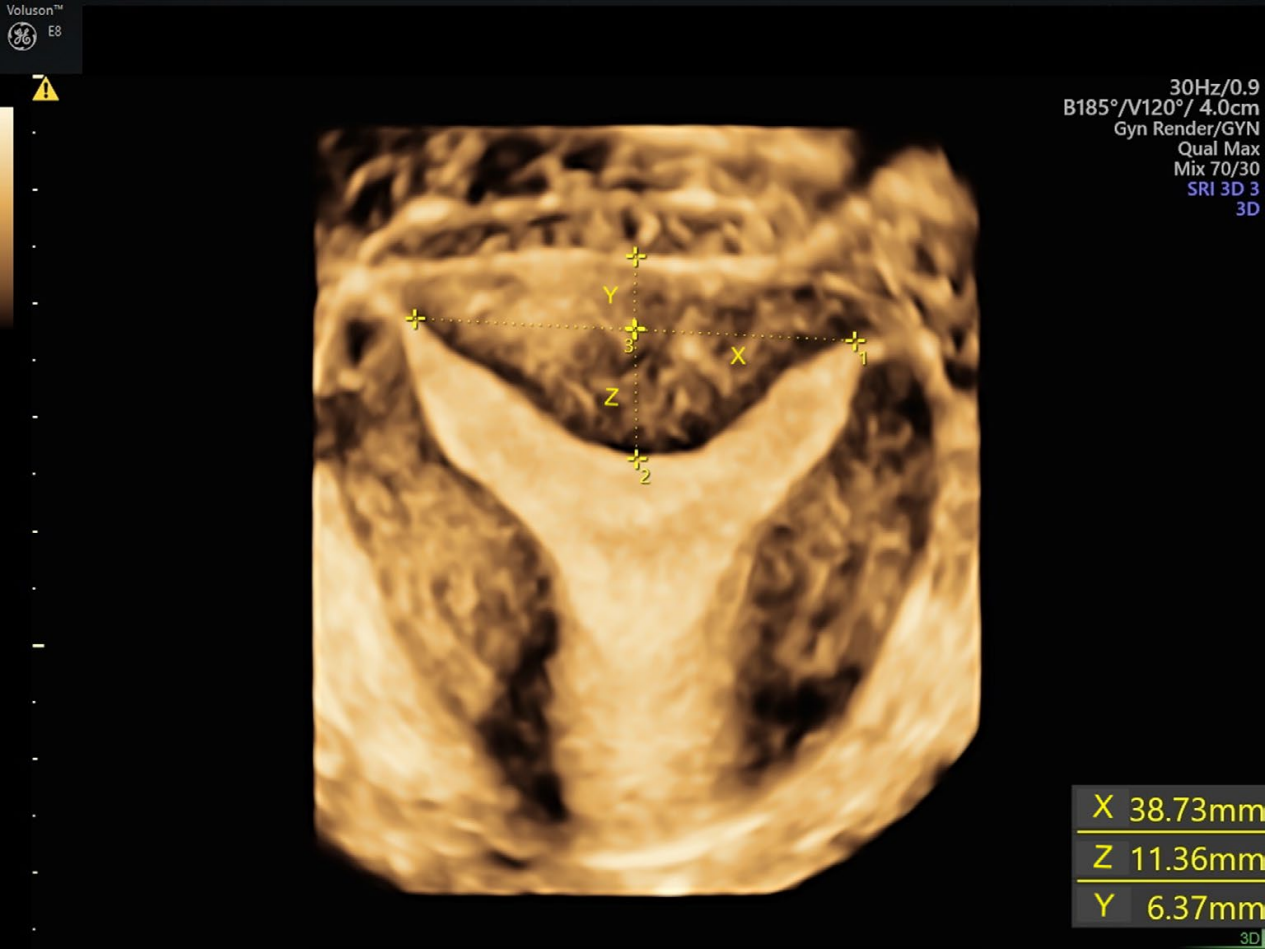
Partial uterine septum (Class U2a according to the ESHRE/ESGE 2013 classification system).

All participants underwent three‐dimensional transvaginal ultrasound (3D‐TVS) performed by experienced operators (A.A. and P.C., two operators with >5 years of experience) during the mid‐luteal phase of the menstrual cycle (days 21–25), which is considered the optimal phase for assessing the uterine cavity using a Voluson E8 Expert ultrasound system (GE Healthcare Ultrasound, Milwaukee, WI, USA) equipped with a multifrequency (4–9 MHz) volumetric transvaginal probe with the possibility of three‐dimensional (3D) study.^10^ The measurements and evaluation of the uterine fundus profile were conducted in the midcoronal plane following the methodology of Grimbizis et al. using coronal reconstruction for septum assessment.^11^ The uterine wall thickness (Y) was measured as the distance between the line connecting the tubal ostia (X) and the fundal uterine serosa. Any internal indentation (Z) was measured as the distance between the interostial line (X) and the edge of the indentation (Figure 1).

### 2.4 Surgical intervention

Following the presurgical evaluation, hysteroscopic metroplasty was performed in an outpatient setting during the early follicular phase of the menstrual cycle. No pharmacological preparations were used to minimize endometrial thickness. The procedure was conducted under paracervical anesthesia by administering 16 mL of ropivacaine (7.5 mg/mL) through four injections at the vaginal fornices, positioned at 3, 5, 7, and 9 o'clock on the cervix, targeting the lateral parametria and uterosacral ligaments. Without the need for cervical dilation, a 5‐mm diameter continuous‐flow hysteroscope with a 30° fore‐oblique telescope and a 5Fr operating channel (Office Continuous‐Flow Operative Hysteroscopy “size 5”; Karl Storz, Tuttlingen, Germany) was used. The uterine cavity was distended with a saline solution (0.9% NaCl), controlled through an electronic irrigation and aspiration system (Endomat, Karl Storz, Tuttlingen, Germany).^12^ Continuous flow was set at 200–350 mL/min, with negative pressure suction at 0.2 bar and positive pressure at 80–100 mmHg, maintaining intrauterine pressure at approximately 40 mmHg throughout the procedure.

All surgeries were performed by the same experienced surgeon (P.C., surgeon with >250 hysteroscopic surgeries), following the technique described by Di Spiezio et al., using a novel graduated intrauterine palpator (Karl Storz, Tuttlingen, Germany). The septum incision began at the apex with a straight bipolar electrode in vapor cutting mode (VC3) at 50 W on the electrosurgical generator (Versapoint; Olympus, Hamburg, Germany). The electrode was applied laterally across the septum from apex to base along a median plane. Metroplasty was halted when the palpator was reached and indicated that the incised septum corresponded to presurgical ultrasound measurements, achieving a total fundal uterine wall thickness of 1 cm.^12^ No uterotonics or antibiotics were administered before, during, or after the procedure.

Three‐dimensional TVS was performed on the day of surgery to assess the postoperative uterine cavity and fundal myometrial thickness. In all patients, the uterine cavity showed a regular configuration, with a total fundal myometrial thickness of 1 cm, calculated as the sum of *Y* and the residual *Z* (*Zr*), defined as the distance between the interostial line and the newly reconstructed inner uterine profile. These measurements confirmed complete septum resection.

### 2.5 Follow‐up and outcomes

Three months after the surgical procedure, all patients underwent a follow‐up visit, which included another 3D‐TVS to assess the outcome of the hysteroscopic metroplasty. Patients who required a second surgical procedure for completion were excluded from data analysis. Patients were contacted again at 12 months for a follow‐up evaluation of their symptoms and were asked to complete the same VAS questionnaire used at baseline via telemedicine. Patients who did not respond to follow‐up, had missing data, or were lost to follow‐up were excluded from the study.

### 2.6 Statistical analysis

The sample size was calculated based on the primary outcome of the study. To calculate the required sample size, we referenced the estimates provided by Fedele et al., who reported a reduction in dysmenorrhea of approximately 30% (measured using a linear pain scale) in women with complete or partial uterine septa who underwent metroplasty, considering both hysteroscopic and abdominal approaches.^7^ Given that our study focused solely on patients with partial uterine septa undergoing hysteroscopic metroplasty, we conservatively estimated a 15% reduction in symptoms, accounting for methodological differences between the studies.

Assuming a correlation coefficient of 0.7, a sample size of 33 patients was calculated to detect a 15% reduction in dysmenorrhea with 80% statistical power and a significance level of 5%. We also projected a 37% patient loss rate due to ongoing pregnancies, based on a recent meta‐analysis of live birth rates in women with uterine septa and a history of primary infertility.^5^ This rate is expected to increase if follow‐up extends beyond 1 year. Additionally, a 10% dropout rate was anticipated due to the need for a second surgical intervention, as reported by Rikken et al.,^13^ and a further 10% loss was expected from non‐adherence to the follow‐up protocol. Consequently, the total estimated sample size required was 52 patients.

Continuous data were expressed as mean ± standard deviation (SD) or median (interquartile range), depending on distribution. We selected statistical tests based on the type and distribution of the data. All statistical analyses were pre‐specified. Normality of continuous variables was assessed using the Shapiro–Wilk test. Categorical variables were reported as frequencies and percentages. Statistical comparisons were conducted using Student's *t*‐test or Mann–Whitney *U*‐test for continuous variables, and the *χ* ^2^ or Fisher's exact test for categorical variables, as appropriate. Spearman's correlation was used to evaluate associations between non‐normally distributed continuous variables or ordinal variables. This approach ensures that statistical analyses are appropriate for the scale and distribution of the data, minimizing the risk of incorrect inferences. Extreme values were excluded after verification of data entry errors. A *P*‐value <0.05 was considered statistically significant in all analyses. Statistical analysis was performed using SPSS software, version 29.0.2.0 (IBM, Armonk, NY, USA).

## 3 RESULTS

Between March 2022 and September 2023, 52 symptomatic patients diagnosed with partial uterine septa and a history of infertility, recurrent pregnancy loss (RPL) or desiring pregnancy underwent hysteroscopic metroplasty. Of the 52 enrolled patients, eight were excluded based on predefined criteria (alternative causes of pelvic pain, incomplete septum resection requiring additional surgery, lost during follow‐up, or pregnancy/hormonal therapy within the defined timeframe), resulting in a final analytic cohort of 44 patients. No stratification into subgroups was performed; all analyses were conducted on the cohort as a single group. No randomization, matching, or allocation procedures were used, as all eligible participants received the same surgical intervention.

**FIGURE 2:**
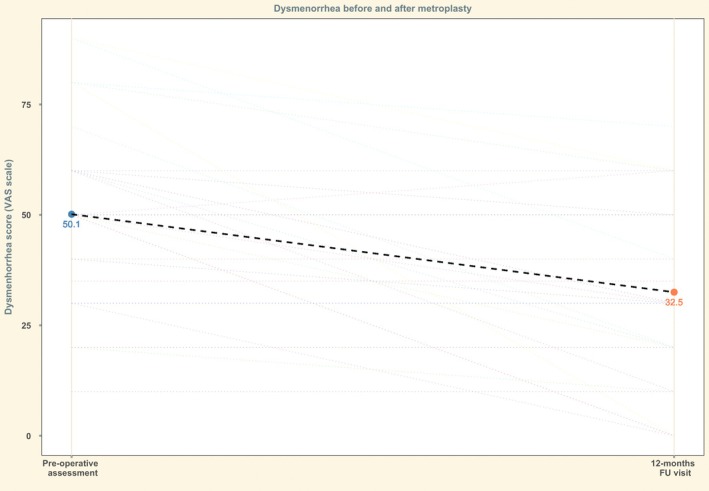
Variation of reported dysmenorrhea before and after hysteroscopic metroplasty.

Data from 44 patients were included in the final analysis. Among these, 34 patients (77%) reported preoperative dysmenorrhea, 14 (33%) reported dyspareunia, and 10 (23%) reported heavy menstrual bleeding. Prior to hysteroscopic metroplasty, the mean dysmenorrhea score on the VAS (ranging from 0 to 100) was 50.1 ± 20.5 (mean ± SD). At the 12‐month follow‐up, the mean dysmenorrhea score had significantly decreased to 32.5 ± 20.2, representing a 35% reduction in pain (mean difference ± standard error [SE]: −17.6 ± 3.75, *P* < 0.001) (Figure 2).

We evaluated the impact of septum length, defined as the internal *Z* indentation in millimeters, on dysmenorrhea outcomes following surgical correction. Spearman correlation analysis demonstrated an 8% correlation between septum length and changes in dysmenorrhea pre‐ and post‐metroplasty, which was not statistically significant (*P* = 0.6). Patients who experienced an improvement in dysmenorrhea had a mean initial *Z*‐value of 11.3 ± 4.9 mm, compared to 9.8 ± 3.8 mm in those without improvement. An independent *t*‐test comparing these means showed a positive difference of 1.6 ± 1.5 mm, but this was not statistically significant (*P* = 0.15).

**FIGURE 3:**
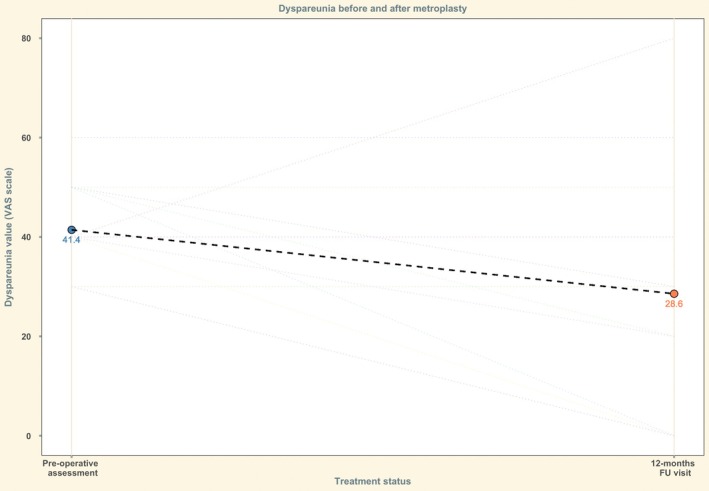
Variation of reported dyspareunia before and after hysteroscopic metroplasty.

Regarding dyspareunia, the mean preoperative score was 41.4 ± 9.5, which decreased to 28.6 ± 24.5 after hysteroscopic metroplasty. This reduction was found to be statistically significant (mean difference ± SE: 12.9 ± 6.2, *P* < 0.028) (Figure 3).

Finally, no statistically significant differences were observed in menstrual flow intensity before and after the hysteroscopic metroplasty procedure (mean ± SD before and after the procedure: 43.7 ± 20.6 vs. 41.7 ± 23.6, *p* = 0.492).

## 4 DISCUSSION

Our study suggests that hysteroscopic metroplasty significantly reduces pain‐related symptoms, in particular dysmenorrhea and dyspareunia, in symptomatic women with partial uterine septa and a history of infertility or RPL or desiring pregnancy. However, we did not observe a significant correlation between symptom improvement and the length of the septum, nor did we find any significant changes in menstrual blood flow before and after the surgical procedure.

Accurate diagnosis and optimal management of patients with uterine septa remain critical challenges, as these conditions can profoundly affect reproductive outcomes and quality of life. Advances in diagnostic techniques, including 3D ultrasound and magnetic resonance imaging, have improved the ability to identify uterine anomalies with greater precision. Concurrently, minimally invasive surgical techniques, such as hysteroscopic metroplasty, have enhanced treatment options available for these patients, providing an improvement in reproductive outcomes.^5^, ^11^, ^14^ Despite these advancements, further research is necessary to refine management strategies and optimize patient care. Hysteroscopic septum resection has long been the standard of care to improve reproductive outcomes, with the primary goal being the removal of the medial septum to restore a regular uterine cavity morphology. Nevertheless, this treatment remains debated, as current evidence is mostly based on observational and non‐randomized comparative studies.^4^

Beyond the issue of infertility, our study aims to evaluate the impact of hysteroscopic metroplasty on the quality of life in symptomatic patients with a partial uterine septum and to explore the potential association between the extent of the uterine septum and symptoms variation. Our results are comparable to those of the study by Fedele et al., which reported that metroplasty is effectively associated with a reduction in dysmenorrhea in patients with a septate uterus.

While the findings of Fedele et al. align with our results, direct comparison is limited due to key differences in study design. Their analysis included two distinct surgical techniques (abdominal and hysteroscopic metroplasty), the use of postoperative corticosteroids, and the inclusion of abdominal metroplasty, a procedure not performed at our center. Additionally, despite their study involving a larger population, only a subset is directly comparable to our cohort, given the inclusion of asymptomatic patients and the variation in surgical approaches.^7^

A deeper understanding of why symptom relief occurs from a pathophysiological standpoint is essential. The histological composition and vascularization of uterine septa remain subjects of ongoing debate in the literature. Rikken et al. propose that the septum consists of endometrium and myometrium similar to the normal uterine wall, while studies by Fascilla et al. and Sparac et al. suggest that the septum has a complex structure, with irregularly arranged muscle fibers resembling myometrial tissue. These studies hypothesize that this atypical muscular structure might contribute to abnormal uterine contractility, potentially explaining the pain symptoms observed in our cohort.^15^, ^16^, ^17^ A previous pilot study from our center demonstrated that septum removal might enhance uterine fundus remodeling by improving the coordination and regularity of uterine contractions. These mechanisms could explain our findings, supporting the notion that hysteroscopic metroplasty alleviates pain symptoms by restoring normal uterine function.^18^ Consequently, the observed relief from pain and the subsequent improvement in quality of life suggest that hysteroscopic metroplasty might offer benefits beyond infertility treatment by restoring normal uterine contractility.

However, symptom relief could also be influenced by potential confounding factors. The placebo effect, concurrent lifestyle modifications, or undiagnosed comorbidities might contribute to the observed improvements. Future studies should account for these variables to strengthen the evidence for a direct causal relationship.

To the best of our knowledge, this study is the first to evaluate the symptomatic relief provided by hysteroscopic metroplasty, specifically for women with partial uterine septa (U2a class). It also explores the relationship between clinical and ultrasonographic characteristics and symptom variation, thereby expanding the scope of treatment indications for this condition.

Despite the promising results of our prospective study, some limitations must be acknowledged. The single‐center nature of the study might restrict the generalizability of our findings, as the patient population might not fully represent the broader diversity seen in multicenter studies. Differences in demographic and clinical characteristics across various populations could influence treatment outcomes. Future research involving multiple centers and more diverse patient cohorts would help confirm the reproducibility of our findings.

Further, the absence of a control group limits our ability to establish a direct causal relationship between hysteroscopic metroplasty and symptom relief. While our findings are in line with previous studies, alternative study designs, such as randomized controlled trials (RCTs), would provide a higher level of evidence by minimizing potential biases. Future RCTs comparing hysteroscopic metroplasty with conservative management or sham procedures could clarify the true impact of surgery on symptom relief and reproductive outcomes.

Additionally, the lack of a standardized hysteroscopic technique across institutions remains a challenge in interpreting surgical outcomes. Although our study was conducted by a single experienced operator using an approach consistent with established techniques in the literature, variations in surgical skill and technique could affect the reproducibility of results. Establishing standardized surgical protocols and postoperative care guidelines would enhance comparability across studies.

Finally, the clinical implications of our findings should be emphasized, particularly in preoperative counseling. Identifying which patients are most likely to experience symptom relief after metroplasty could enhance shared decision‐making and set more realistic patient expectations. Future research should focus on long‐term outcomes, assessing whether symptom improvement is sustained over time or if relapses occur.

## AUTHOR CONTRIBUTIONS

In accordance with the ICMJE authorship criteria, all authors have made significant contributions to the study as outlined below: **Conceptualization and study design:** A.A., A.V., C.F., E.B., E.M., G.C. **Data collection:** C.F., E.M., E.B., P.DB., R.S., A.V., G.C. **Data analysis and interpretation:** A.A., E.M., P.C. **Original draft, review and editing:** A.A., C.F., E.B., A.V., E.M., P.DB., G.C., R.S., P.C. **Supervision and project administration:** A.A., A.V., R.S., P.C. All authors have read and approved the final manuscript and have agreed to be accountable for the accuracy and integrity of the entire work.

## FUNDING INFORMATION

None.

## CONFLICT OF INTEREST STATEMENT

None.

## Supporting information


Appendix S1.

Appendix S2.

## Data Availability

Research data are not shared.
