# Supplementary material for: Hysteroscopy metroplasty for partial septate uterus: Just a matter of infertility?
Source: Int J Gynaecol Obstet. 2026 Feb 24;174(2):906–12. doi: 10.1002/ijgo.70906 (PMC13377344; doi:10.1002/ijgo.70906)
Supplement: Supplementary file 2 — Appendix S2. [file IJGO-174-906-s001.docx]

**No. 552/2024/Oss/AOUBo, reviewed on 12/09/2024**

NOTE: For any further correspondence regarding the study in question, it is essential to reference the internal CE code identifying the study mentioned above and the date of the review session.

This opinion is issued by the local Secretariat at IRCCS Azienda Ospedaliero–Universitaria di Bologna, Policlinico di Sant’Orsola (study contact: Scientific Secretariat). All communications regarding this opinion must be sent to the email address cometico@aosp.bo.it.

**To:** University of Bologna - Alma Mater Studiorum sam.studiclinici@unibo.it

**Cc:** Prof. Paolo Casadio
UO Gynecology and Human Reproductive Physiology
IRCCS Azienda Ospedaliero–Universitaria di Bologna
paolo.casadio@aosp.bo.it

Prof. Renato Seracchioli
Director, UO Gynecology and Human Reproductive Physiology
IRCCS Azienda Ospedaliero–Universitaria di Bologna
renato.seracchioli@aosp.bo.it

**Subject:**Transmission of Opinion on Study:
“Evaluation of Variations in Ultrasound Measurements and Quality-of-Life Outcomes in Patients Undergoing Operative Hysteroscopy”

**Internal CE Code:**552/2024/Oss/AOUBo
**Protocol Code:**HystUS
**Sponsor:**University of Bologna - Alma Mater Studiorum
**Principal Investigator:**Prof. Paolo Casadio
**Clinical Center:** UO Gynecology and Human Reproductive Physiology - Seracchioli - AOUBo

**Documentation Reviewed During the Session:**

Study Protocol, v.1 dated 19/06/2024

Study Protocol Synopsis, v.1 dated 19/06/2024

CRF, v.1 dated 19/06/2024

Letter of Intent, dated 26/06/2024

Study-Specific Questionnaire, v.1 dated 19/06/2024

Local Feasibility Assessment, signed by PI Dr. Casadio on 19/06/2024

CEAVEC opinion on the HystUS study (subsequently withdrawn), with CE code 205.2020.Oss.AOUBo

Principal Investigator's Curriculum Vitae, dated 09/05/2024

Conflict of Interest Declaration, signed by PI Dr. Casadio on 09/05/2024

Participant Information and Study Consent Form, v.1 dated 19/06/2024

Data Privacy Information and Consent Form, v.1 dated 19/06/2024

**Committee Decision:**During the telematic session held on 12/09/2024, the Ethics Committee unanimously expressed a favorable opinion for the conduct of the study.

**Conditions for Study Initiation:**

The study may commence only after obtaining formal authorization from the General Management of the relevant healthcare facility, in accordance with Article 7 of Regional Law No. 9/2017.

To facilitate monitoring of the study's progress, the Principal Investigator is required to provide periodic progress reports to the Ethics Committee from the study’s initiation until its conclusion. Reports should be submitted annually or more frequently in cases of significant events affecting the study's conduct and/or increasing risk to participants.

To this end, the monitoring form available on the Ethics Committee’s website (<https://www.aosp.bo.it/sites/default/files/Allegati_ricerca/modulo_monitoraggio_ceavec.docx>) should be completed, signed, and dated. The completed form must be sent exclusively in electronic format to the dedicated email address monitoraggiostudi@aosp.bo.it, with the subject line including the name of the clinical center, the Principal Investigator’s name, and the study code assigned by the Committee.

Best regards,
THE PRESIDENT
*(Prof. Elisabetta Poluzzi)*

**Attachment**: List of Ethics Committee members present during the issuance of this opinion.

| **Name** | **Role as per DM 30 January 2023** | **Affiliated Institution** | **Present/Absent** |
| --- | --- | --- | --- |
| Elisabetta Poluzzi | Pharmacologist | University of Bologna | Present |
| Primiano Iannone | Clinical Expert in Gastroenterology | AUSL of Bologna | Absent |
| Federica Banorri | Legal Expert | IRCCS AOU of Bologna | Absent |
| Francesca Bonetti | Representative of patient/citizen associations on health matters | AGD (Young Diabetics Association) | Present |
| Elisabetta Cagni | Medical Physicist | AUSL of Romagna | Present |
| Fausto Catena | Clinical Expert in General Surgery | Independent Practitioner | Present |
| Paolo Cristiani | Clinical Expert in Gynecology and Obstetrics | Retired | Absent |
| Alessandra De Palma | Forensic Doctor | Independent Practitioner | Present |
| Danilo Di Diodoro | Clinical Expert in Psychiatry | AOU of Parma | Absent |
| Giuseppe Di Pasquale | Clinical Expert in new diagnostic and therapeutic techniques (invasive/semi-invasive) | University of Bologna | Present |
| Susanna Esposito | Pediatrician | IRCCS AOU of Bologna | Present |
| Carla Faralli | Bioethics Expert | Retired | Present |
| Simona Ferrari | Genetics Expert | Independent Practitioner | Present |
| Graziella Filippini | Clinical Expert in Neurology | IRST "Dino Amadori" - IRCCS | Absent |
| Carlo Finelli | Clinical Expert in Hematology | IRCCS Rizzoli Orthopedic Institute of Bologna | Absent |
| Lorenzo Gasperoni | Pharmacist (SSN) | AUSL of Imola | Present |
| Monica Guberti | Representative of Health Professions | University of Bologna | Present |
| Carlo Impallomeni | Clinical Expert in Orthopedics and Traumatology | AUSL of Reggio Emilia | Present |
| Lamberto Manzoli | Biostatistician | IRCCS AOU of Bologna | Present |
| Gianluca Mazza | Insurance Expert | IRST "Dino Amadori" - IRCCS | Absent |
| Michela Pastore | General Practitioner | MMG affiliated with AUSL Ferrara | Present |
| Elisabetta Sanvito | Medical Devices Expert | Retired | Present |
| Emanuela Scarpi | Biostatistician | IRST "Dino Amadori" - IRCCS | Present |
| Patrizia Serra | Nutrition Expert | IRST "Dino Amadori" - IRCCS | Present |
| Savino Spadaro | Expert in Anesthesia and Resuscitation | AOU of Ferrara | Present |
| Marcello Tiseo | Oncology Expert | AOU of Parma | Absent |

The following representatives from the Secretariats participated in the session (non-voting members):

- Stefania Proni, Giacomo Chiabrando, Marta Sperti, Alessandra Brunetti, Serena Di Rienzo: Central Secretariat and Local Secretariat at IRCCS - Azienda Ospedaliero Universitaria di Bologna.
- Corrado Iacono, Giulia Tempesta: Local Secretariat at Azienda USL di Bologna and Imola.
- Simonetta Gamberini, Arianna Ferretti Cherkes Zade: Local Secretariat at the Rizzoli Orthopedic Institute.
- Marco Voci: Local Secretariat at Azienda Ospedaliero Universitaria di Ferrara.
